# Multifunctional Polymer-Modified P-CaO_2_@Au@OVA@Cu@DHPs Nanoparticles Enhance SARS-CoV-2 mRNA Vaccine-Induced Immunity via the cGAS–STING Signaling Pathway

**DOI:** 10.3390/polym17192636

**Published:** 2025-09-30

**Authors:** Yanle Zhi, Shengchao Wang, Haibo Zhang, Guimin Xue, Zhiqiang Zhang

**Affiliations:** 1 Academy of Chinese Medical Sciences, Henan University of Chinese Medicine, Zhengzhou 450045, China; 2 Collaborative Innovation Center of Research and Development on the Whole Industry Chain of Yu-Yao, Henan University of Chinese Medicine, Zhengzhou 450046, China; 3 Engineering Technology Research Center of Traditional Chinese Medicine Health Industry in Henan Province, Zhengzhou 450046, China

**Keywords:** dendrobium huoshanense polysaccharide, nanoparticles, vaccine adjuvant, immune evaluation

## Abstract

The success of mRNA-based SARS-CoV-2 vaccines has been confirmed in both preclinical and clinical settings. However, the development of safe and efficient mRNA vaccine delivery platforms remains challenging. In this report, PBAE-G-B-SS-modified CaO_2_ nanofibers and Au@OVA@Cu@Dendrobium huoshanense polysaccharides were employed to establish novel self-assembling polymeric micelles (CaO_2_@Au@OVA@Cu@DHPs) capable of serving as both an adjuvant and a delivery system for mRNA vaccines. In vitro, CaO_2_@Au@OVA@Cu@DHPs nanoparticles (NPs) were conducive to effective macrophage antigen uptake and efficient antigen processing. In vivo, P-CaO_2_@Au@OVA@Cu@DHPs NP administration was associated with a reduction in the ovalbumin (OVA) release rate that was conducive to the sustained induction of long-term immunity and to the production of higher levels of different IgG subtypes, suggesting that these effects were attributable to enhanced antigen uptake by antigen-presenting cells. Overall, these present data highlight the promise of these P-CaO_2_@Au@OVA@Cu@DHPs NPs as an effective and safe platform amenable to vaccine delivery through their ability to provide robust adjuvant activity and sustained antigen release capable of eliciting long-term immunological memory while potentiating humoral and cellular immune responses.

## 1. Introduction

The ongoing COVID-19 pandemic, caused by SARS-CoV-2, represents a persistent and severe threat to global economic stability and public health [1], with the circulating Omicron variants (BF.7, BQ.1, XBB) having emerged as the predominant circulating version of SARS-CoV-2 after acquiring several advantageous mutations, contributing to extremely high rates of infection [2,3]. The BF.7 Omicron variant has been reported to exhibit the most efficient ability to evade host immunity of any variant documented to date [4,5].

Vaccination is a safe and effective means of preventing disease through the induction of cellular and humoral immunity [6], with common vaccine types including live-attenuated, inactivated, toxoid, and subunit vaccines [7]. Modern approaches to vaccine preparation have led to a growing interest in safer protein subunit and recombinant protein-based vaccines over more traditional vaccination strategies [8]. Relative to inactivated or live-attenuated vaccines [9], these subunit vaccines targeting defined antigens are not subject to the same safety concerns and limitations but are generally less immunogenic [10]. It is thus crucial that appropriate immunostimulatory adjuvants be employed to enhance the efficacy of these subunit vaccines [11]. Aluminum-based adjuvants have long been used in this context [12], but they induce relatively weak cellular and humoral immune responses that hamper their durable clinical utility [13], in addition to inducing disruptive side effects [14] and serving as inefficient adjuvants for certain antigens [15]. There thus remains a pressing need for the development of safer and more effective vaccine adjuvants.

Plants in the Dendrobium genus (family Orchidaceae) have long been applied for medicinal purposes in Asian nations [16]. *Dendrobium huoshanense* (*D. huoshanense*), for example, is often used to prepare soups and teas purported to exhibit cardioprotective, antipyretic, and immune-enhancing activities [17]. *D. huoshanense* is composed of a variety of bioactive polysaccharides, alkaloids, and aromatic compounds, with the health benefits of *D. huoshanense* having primarily been attributed to polysaccharides that have documented antioxidant, antitumor, and immunomodulatory properties [18]. In our previous study, we determined that *D. huoshanense* polysaccharide (DHP) extracts consist of glucose (65.04%), mannose (14.23%), galactose (8.17%), galacturonic acid (6.41%), rhamnose (2.34%), and xylose (1.25%), and that these DHPs can enhance immune responses. These DHPs have also been reported to enhance the secretion of cytokines capable of promoting cellular and humoral immunity including interleukin (IL)-4, IL-6, and tumor necrosis factor (TNF)-α when used to treat murine RAW264.7 macrophages [19]. We have also previously applied DHPs as an adjuvant capable of enhancing immune activity [20]. As DHPs are water-soluble, however, they are rapidly metabolized and exhibit limited untargeted bioactivity, limiting their application as a vaccine adjuvant.

Nanodrug delivery systems have emerged as a promising vaccine adjuvant platform as they have the potential to modulate the biodistribution and pharmacokinetic properties of administered polysaccharides in a manner conducive to more robust targeted cellular and humoral immunity [21]. These nanomaterials can also recapitulate pathogen-associated structural characteristics detected by the immune system [21] and are ideal for the simultaneous delivery of antigens and adjuvants to provoke immune signaling responses and improve the bioavailability of administered drugs [22]. Accordingly, adjuvant-based nanoparticles (NPs) can enable vaccine development through the incorporation of adjuvant polysaccharides, thereby shielding them from degradation.

Gold is rich in trace elements such as copper and zinc, which are essential for maintaining the normal physiological functions of the body. Therefore, consuming gold can supplement these trace elements. Copper and zinc play significant roles in growth and development, immune system function, and skin health. The levels of copper and zinc in the body can be increased through food intake or oral supplements. Some components in gold have antioxidant effects, which can eliminate free radicals in the body, reduce the damage caused by oxidative stress to cells, and thereby enhance the body’s disease resistance. Metal-based NPs are commonly used in drug delivery applications [23], as they can be easily prepared, remain stable in storage, exhibit good biocompatibility, are highly versatile, and have a good safety profile [24]. These NPs can be designed for targeted, controlled drug delivery and for use in particular imaging applications. While the slow metabolic processing of metal NPs limits their clinical utility, the application of a thin polymer surface layer can endow these particles with enhanced affinity, biocompatibility, and biosensing properties [25]. CaO_2_ NPs, in particular, offer promise as candidates for antigen or drug delivery applications [26], exhibiting desirable biodegradation and biocompatibility profiles such that they have been employed as pharmaceutical excipients for oral drug and vaccine delivery [27]. CaO_2_ is sensitive to acidic conditions such as those found within endolysosomal compartments, enabling the release of CO_2_ NP-encapsulated drugs or antigen cargo in particular biological settings [28]. Unmodified CaO_2_-coated metal NPs, however, generally present with poor water solubility that hampers adjuvant activity. Poly (beta-amino esters) (PBAEs) are nontoxic, pH-sensitive, structurally diverse drug carriers that are easily synthesized [29]. PBAEs contain many ester bonds that are readily hydrolyzed in vivo prior to eventual excretion [30], and PBAE-coated NPs can enable more robust cellular uptake and immunogenicity when leveraged as a drug delivery platform.

**Scheme 1:**
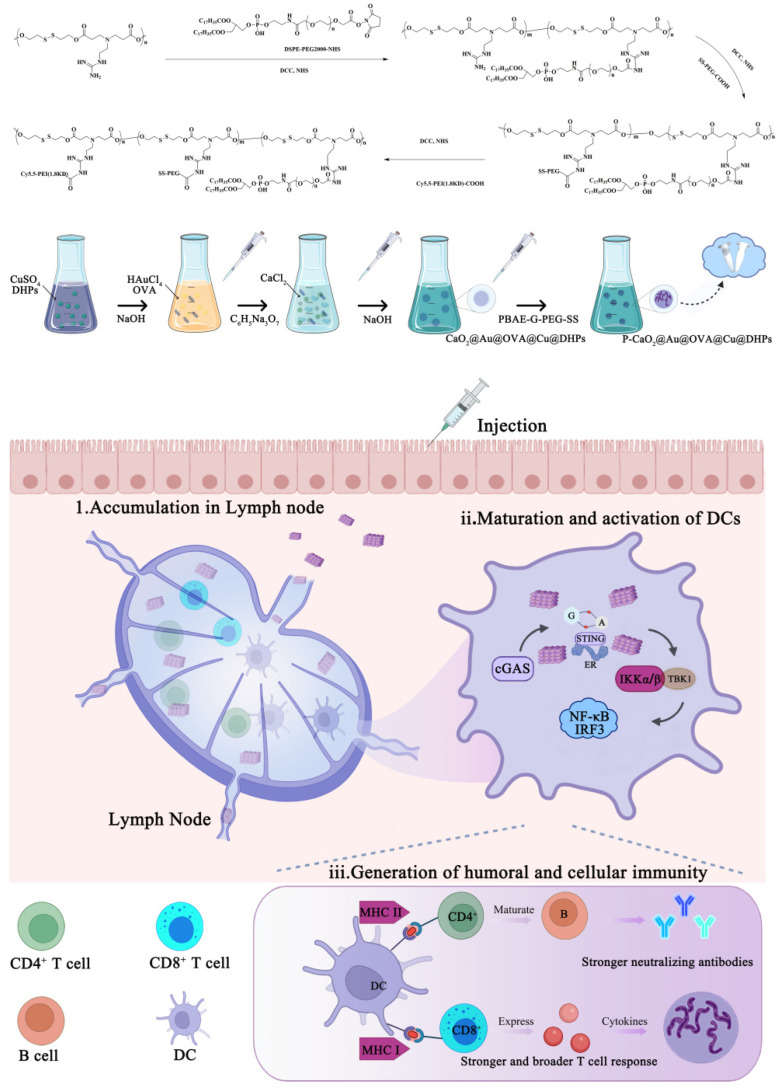
The schematic illustration of P-CaO_2_@Au@OVA@Cu@DHPs mediated activation of immune system via regulate cGAS-STING pathway.

We have previously shown that incorporating ovalbumin (OVA) into DHP CaCO_3_ NPs as a vaccine delivery platform can stimulate robust cellular and humoral immunity, but our preliminary experiments suggested that the immunostimulatory effects of DHPs were limited when using these DHPs@CaCO_3_ NPs [31]. In this study, we thus developed a CaO_2_-modified hybrid NP structure consisting of Au, OVA, Cu, and DHPs (CaO_2_@Au@OVA@Cu@DHPs). A microfluidic mixing device was used to incorporate OVA and DHPs into NPs, and the biological activity and biocompatibility of these NPs were enhanced by multifunctional PBAE-G-PEG-SS polymer modification (Scheme 1). We hypothesized that these P-CaO_2_@Au@OVA@Cu@DHPs NPs would serve as an effective vaccine delivery and adjuvant platform capable of targeting and activating antigen-presenting cells (APCs), thereby inducing robust humoral and cellular immunity. Following the physicochemical characterization of the prepared NPs, their ability to induce macrophage activation and antigen uptake was assessed. These P-CaO_2_@Au@OVA@Cu@DHPs NPs were then combined with the SARS-CoV-2 mRNA vaccine to assess the induction of humoral and cellular immunity in vivo.

## 2. Materials and Methods

### 2.1. Materials

DHPs (95%, CY200618) were obtained from Professor Bingji Ma of Henan Agricultural University. Anhydrous CaCl_2_, NaOH, and H_2_O_2_ (30%) were obtained from Beijing Chemical Works (Beijing, China). Copper chloride dihydrate (CuCl_2_·5H_2_O) was obtained from Xilong Chemical Reagent Co., Ltd. (Shantou, China). Succinimidyl Succinate-Polyethylene glycol–COOH (SS-PEG-COOH, Item No. R-N-0027) was obtained from Xian Ruixi Biological Technology Co., Ltd. RPMI-640, fetal bovine serum (FBS), 3-[4,5-dimethylthiazol-2-yl]-2,5-diphenyltetrazolium bromide (MTT), 0.25% trypsin-EDTA, JC-1, and an Annexin V FITC/PI kit were obtained from Thermo Fisher Scientific (Shanghai, China). Rhod-2 AM and MitoTracker Red were obtained from Yeasen (Shanghai, China). The compound 2′,7′-dichlorofluorescin diacetate (DCFH-DA) was obtained from Bestbio (Shanghai, China). Alizarin red was obtained from Yunaye (Shanghai, China). Murine TNF-α (Tianjin, China) and IFN-γ (Tianjin, China) ELISA kits were obtained from Anoric Biotechnology Co., Ltd. (Tianjin, China). Other reagents were of analytical grade and used without further modification.

### 2.2. Methods

#### 2.2.1. *D. huoshanense* Polysaccharide Preparation and Characterization

DHPs were extracted, isolated, purified, and characterized as in our prior study [32].

#### 2.2.2. Au@OVA@Cu@DHPs Synthesis

A combination of 5 mL of aqueous CuSO_4_·5H_2_O (10 mg/mL) and a 10 mL aqueous DHP-containing solution was mixed for 5 min at room temperature, after which the pH of the solution was adjusted to 10.0, followed by stirring for a further 24 h. The resultant Cu@DHPs were then collected via centrifugation, rinsed three times using deionized water, and freeze-dried. Next, 10 mL of an aqueous Cu@DHPs solution (10 mg/mL) was mixed with 10 mL of aqueous OVA (10 mg/mL) for 5 min at room temperature, followed by the addition of aqueous AuCl_4_H (1 M), with this solution then being stirred for an additional 30 min at 150 °C. Aqueous NaCl was then added to this solution, which was stirred for an additional 15 min. The resultant Au@OVA@Cu@DHPs were then collected via centrifugation, rinsed three times using deionized water, and freeze-dried.

#### 2.2.3. CaO_2_-Au@OVA@Cu@DHPs Synthesis

A mixture composed of 5 mL of aqueous NaHA (5 mg/mL), 2.5 mL of aqueous CaCl_2_ (0.1 M), and 2.5 mL of aqueous Au@OVA@Cu@DHPs was stirred for 5 min at room temperature, followed by the addition of 5 mL of aqueous NaOH (0.25 M) and stirring for an additional 5 min, after which the dropwise addition of 200 μL of H_2_O_2_ (30%) was performed with continuous stirring at room temperature for an additional 30 min. The product was then collected via centrifugation, rinsed three times using deionized water, and freeze-dried.

#### 2.2.4. PBAE-G-PEG-SS Preparation

As shown in Scheme 1, PBAE-G-PEG-SS conjugate synthesis was performed through a three-step process. First, an amidation reaction took place between the PBAE-G amino groups and the DSPE-PEG_2000_-NHS carboxyl groups. Then, an amidation reaction took place between the PBAE-G-B amino groups and the SS-PEG-COOH carboxyl groups. Finally, an amidation reaction took place between the PBAE-G-PEG-SS amino groups and the Cy5.5-PEI_1.8KD_-COOH carboxyl groups.

#### 2.2.5. P-CaO_2_-Au@OVA@Cu@DHPs Synthesis

P-CaO_2_-Au@OVA@Cu@DHPs were prepared by suspending 20 mg of CaO_2_-Au@OVA@Cu@DHPs in 10 mL of PBS (pH 8.0) containing 200 mg of polymer components. This solution was then stirred continuously for 8 h, followed by dialysis (MWCO: 4500 Da) against PBS (pH 7.4) to facilitate organic solvent removal. The resultant product was then centrifuged and washed three times using deionized water, after which it was freeze-dried.

#### 2.2.6. Nanoparticle Characterization

A Malvern Zetasizer Nano ZS device (Hydro2000Mu, Malvern Instruments, Malvern, UK) was used to characterize NP particle size, polydispersity index (PDI), and zeta potential values via dynamic light scattering (DLS) analyses. NP morphological characteristics were evaluated through transmission and scanning electron microscopy (TEM and SEM, respectively). DHP and OVA encapsulation efficiency (EE) values were assessed with a previously reported modified microcolumn centrifugation approach [31]. Loading efficiency values were calculated as follows:

DHP loading efficiency = (1 − Q1/Q2) × 100%

where Q1 corresponds to the amount of free DHPs and Q2 corresponds to the total amount of DHPs in P-CaO_2_-Au@OVA@Cu@DHPs suspensions.

OVA loading efficiency = (1 − Q3/Q4) × 100%

where Q3 corresponds to the amount of free OVA and Q4 corresponds to the total amount of OVA in P-CaO_2_-Au@OVA@Cu@DHPs suspensions.

#### 2.2.7. Analyses of DHPs and OVA Release

Initially, 1 mg of P-CaO_2_-Au@OVA@Cu@DHPs was suspended in 2 mL of appropriate solutions, and the solution was stirred for a range of time points. Supernatants were then collected via centrifugation, and DHP and OVA concentrations therein were assessed via a phenol–sulfuric acid method.

#### 2.2.8. Analyses of Particle Stability

NP stability was assessed by incubating NP suspensions at 4 °C for 7, 14, 21, and 28 d, after which particle size, PDI, and zeta potential values were measured. The EE values for these NPs (DHPs-EE and OVA-EE) were measured at the same time points. All analyses were conducted in triplicate.

#### 2.2.9. H_2_O_2_-Responsive Disassembly of P-CaO_2_-Au@OVA@Cu@DHPs

The disassembly of P-CaO_2_-Au@OVA@Cu@DHPs under H2O2 stimulation with different concentrations was demonstrated via transmission electron microscopy (TEM). In brief, P-CaO_2_-Au@OVA@Cu@DHPs (10 μg) was dispersed in PBS solution with the addition of H2O2 (0, 0.5, 5, and 10 mM).

#### 2.2.10. Peritoneal Macrophage Isolation

To isolate peritoneal macrophages, 6-week-old ICR mice were used as described in prior reports. Briefly, 1 mL of 6% starch broth medium was intraperitoneally injected into these mice. Three days later, peritoneal macrophages were harvested via the use of PBS to lavage the peritoneal cavity two times. The lavage fluid was then centrifuged (1500 rpm, 8 min), followed by suspension at 2 × 10^5^ cells/mL in complete culture media. Cells were then incubated in a humidified tissue culture incubator (37 °C, 5% CO_2_) for 4 h, after which PBS was used to wash away nonadherent cells, followed by the addition of fresh complete culture media. The resultant macrophages were used in subsequent experiments.

#### 2.2.11. Macrophage Viability Analyses

An MTT assay was used to assess peritoneal macrophage viability as described in prior reports. Briefly, these cells were added to 96-well plates (100 μL/well), followed by the addition of a range of concentrations of P-CaO_2_-Au@OVA@Cu@DHPs NPs or free DHPs (0–500 μg/mL) in 100 µL. Cells were then cultured for 48 h, after which 30 μL of MTT reagent (5 mg/mL) was added to each well. Following a further 4 h incubation, media was exchanged for DMSO (100 μL), and absorbance at 570 nm was assessed with a microplate reader (Thermo, Waltham, MA, USA). Viability was then calculated based on the ratio of absorbance between the control and experimental groups. All analyses were performed in quadruplicate.

#### 2.2.12. Flow Cytometry

Macrophage surface MHCII, CD86, and CD80 expression levels were assessed via flow cytometry [33]. Macrophages were cultured for 48 h in the presence of P-CaO_2_-Au@OVA@Cu@DHPs or free DHPs (30 μg/mL), after which they were harvested with Trypsin-EDTA, centrifuged (4000 rpm, 5 min), and stained for 30 min with antibodies specific for MHCII, CD80, and CD86 while protected from light. Cells were then rinsed three times with PBS, followed by analysis using a flow cytometer (FACSCalibur, BD Biosciences, San Jose, CA, USA).

#### 2.2.13. Macrophage Cytokine Secretion Analyses

Macrophages were cultured for 48 h in the presence of P-CaO_2_-Au@OVA@Cu@DHPs or free DHPs (30 μg/mL). Levels of IL-2, IL-4, IL-1β, IL-6, IFN-γ, and TNF-α in harvested cell culture supernatants were then assessed with commercial ELISA kits (Multi Sciences, Hangzhou, China), with absorbance at 450 nm analyzed with a microplate reader (Thermo, Waltham, MA, USA). All analyses were performed in triplicate.

#### 2.2.14. Analysis of Macrophage Uptake Activity

Macrophages were incubated for 12 h in 24-well plates in the presence of P-CaO_2_-Au@OVA@Cu@DHPs, OVA-FITC (green), and PBAG-G-PEG-SS (red) solutions. Following this incubation, cells were repeatedly rinsed with PBS prior to Hoechst 33,342 counterstaining for 10 min. After multiple additional PBS washes to remove free PBS, an inverted fluorescence microscope was used to image these cells.

#### 2.2.15. Animal and Vaccination Experiments

All animal experimental procedures were conducted in conformity with institutional guidelines of the Animal Ethics Committee of animal Experiment Center of Henan University of Chinese Medicine (Zhengzhou, China), approval number: DWLLGZR202103038, and followed the National Research Council’s guide for the care and use of laboratory animals.

#### 2.2.16. SARS-CoV-2 Receptor-Binding Domain (RBD) mRNA Vaccine Preparation

The preparation of SARS-CoV-2 RBD mRNA was performed as described in a prior report [33]. The final P-CaO_2_-Au@OVA@Cu@DHPs/mRNA complexes were freshly prepared prior to each round of immunization by mixing a range of P-CaO_2_-Au@OVA@Cu@DHP concentrations with a fixed mRNA stock solution concentration in sterile RNase-free dH_2_O. Samples were briefly vortexed, followed by incubation for 30 min at room temperature to permit particle formation.

#### 2.2.17. Murine Immunization

Female BALB/c mice (6–8 weeks old) were randomly assigned to six groups (*n* = 5/group) and intramuscularly (i.m.) immunized with P-CaO_2_-Au@OVA@Cu@DHPs/mRNA vaccine complexes (5 μg, 10 μg, or 30 μg mRNA/mouse, N/P = 32) at 14-day intervals. Mice in the negative control groups were administered equal volumes of saline, 30 μg of mRNA, and polyethyleneimine copolymer (PVES) on the same immunization schedule. On days 14 and 28 post-immunization, blood was collected from the orbital vein and centrifuged (30 min, 4000 rpm, 4 °C) to collect serum, which was then aliquoted and stored at −20 °C for analyses of RBD-specific IgG titers.

#### 2.2.18. Serum Antibody ELISAs

SARS-CoV-2 RBD-specific IgG titers were assessed using a commercially available ELISA kit (KIT006, Sino Biological, Beijing, China) based on provided directions. Briefly, 96-well plates were coated with recombinant SARS-CoV-2 RBD protein, blocked, and 100 µL of 10-fold serum dilutions (beginning at 1:100) were added to each well. Following a 2 h room temperature incubation, plates were rinsed five times using wash buffer, incubated for 1 h at room temperature with horseradish peroxidase (HRP)-goat anti-mouse IgG (Sino Biological, Beijing, China), rinsed five more times, and a chromogenic substrate solution was added for 20 min at room temperature. Absorbance at 450 nm was then analyzed with a microplate reader (Sunrise, TECAN, Mannedorf, Switzerland). Titer values were calculated based on instructions provided by the manufacturer.

#### 2.2.19. Intracellular Cytokine Staining

Antigen-specific CD4^+^ and CD8^+^ immunity was evaluated through intracellular cytokine staining. Briefly, at 4 weeks after immunization, murine spleens were harvested and splenocytes were purified prior to transfer into 12-well plates (1 × 10^6^/well). These cells were then stimulated for 2 h with a peptide pool (2 μg/mL of individual peptides). Then, cells were incubated for 4 h in the presence of Golgiplug (1 μL/mL; BD Biosciences), followed by staining with antibodies specific for CD4 and CD8 (Biolegend). Cells were then fixed and permeabilized with an appropriate permeabilization buffer (BD Biosciences), followed by staining using anti-IFN-γ and anti-IL-4 (Biolegend). A BD FACSAria II flow cytometer was used for flow cytometry analyses, with FlowJo 10.0 being used to analyze the resultant data.

#### 2.2.20. Serum Cytokine Analyses

Serum IFN-γ, TNF-α, IL-4, and IL-6 levels were assessed on days 21 and 28 following primary vaccination using commercial sandwich ELISA kits (Multi Sciences) based on provided directions. Samples were analyzed in quadruplicate, and absorbance values were assessed at 450 nm.

#### 2.2.21. Statistical Analysis

Data are means ± standard error of the mean (SEM) and were compared with Duncan and LSD multiple range tests. *p* < 0.05 served as the threshold to define significance.

## 3. Results

### 3.1. DHP Isolation, Purification, and Physicochemical Characterization

**Figure 1:**
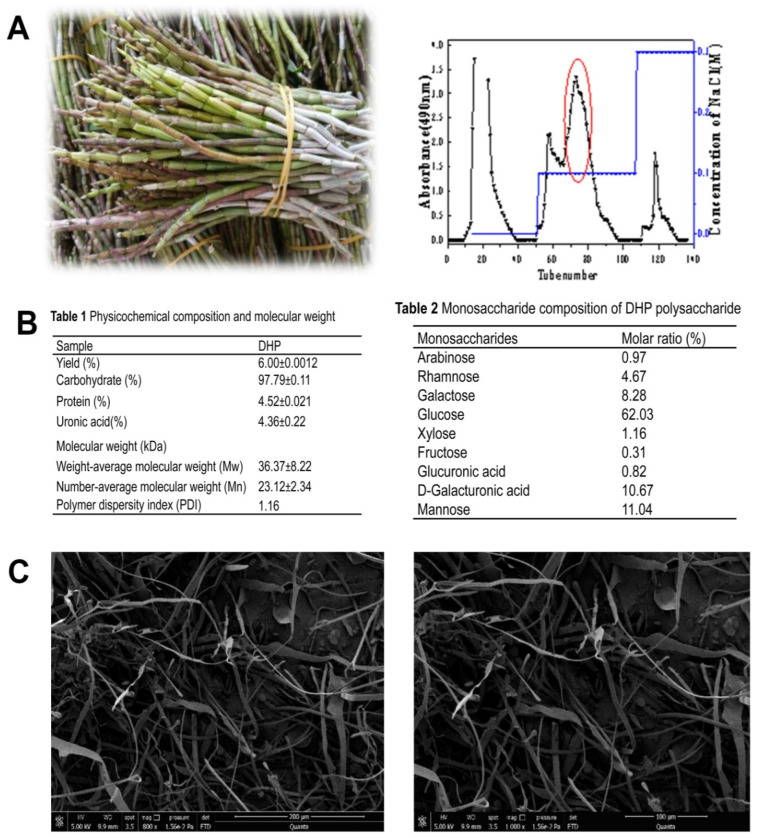
(**A**): Images of *D. huoshanense* and elution profiles for crude polysaccharide preparations separated with a DEAE-cellulose column, together with the elution profiles for three Sephadex G-150 column eluent fractions and the structural characteristics of the purified fraction. (**B**): Sample monosaccharide composition and physicochemical characteristics. (**C**): DHP was imaged via SEM (800×; 1000×).

A subcritical water extraction approach was used to extract DHPs, achieving an overall yield of 6% relative to the dry weight of the raw input material. Following deproteinization and decolorization, DHPs were fractionated and purified using a DEAE-52 column and Sephadex G-100 chromatography. DEAE-52 column purification yielded four fractions, including a deionized water fraction, two 0.1 M NaCl fractions, and one 0.3 M NaCl fraction (Figure 1). The second of the 0.1 M NaCl fractions (Section 2), with a 6% DHP yield, was purified through Sephadex G-100 chromatography, producing a single symmetrical peak designated DHPs-2 with an 89.3% yield. The respective average molecular weight (Mw) and number-average Mw (Mn) values for the prepared DHPs-2 fraction were 36.37 × 10^3^ and 23.12 × 10^3^ Da. The DHPs-2 preparation exhibited a PDI of ~1.16, with this value being close to 1 and consistent with a homogeneous fraction. The DHPs-2 sample was primarily composed of glucose (62.03%), mannose (11.04%), galactose (8.28%), galacturonic acid (10.67%), rhamnose (4.67%), and xylose (1.16%) (Figure 1).

### 3.2. P-CaO_2_-Au@OVA@Cu@DHPs Synthesis and Characterization

**Figure 2:**
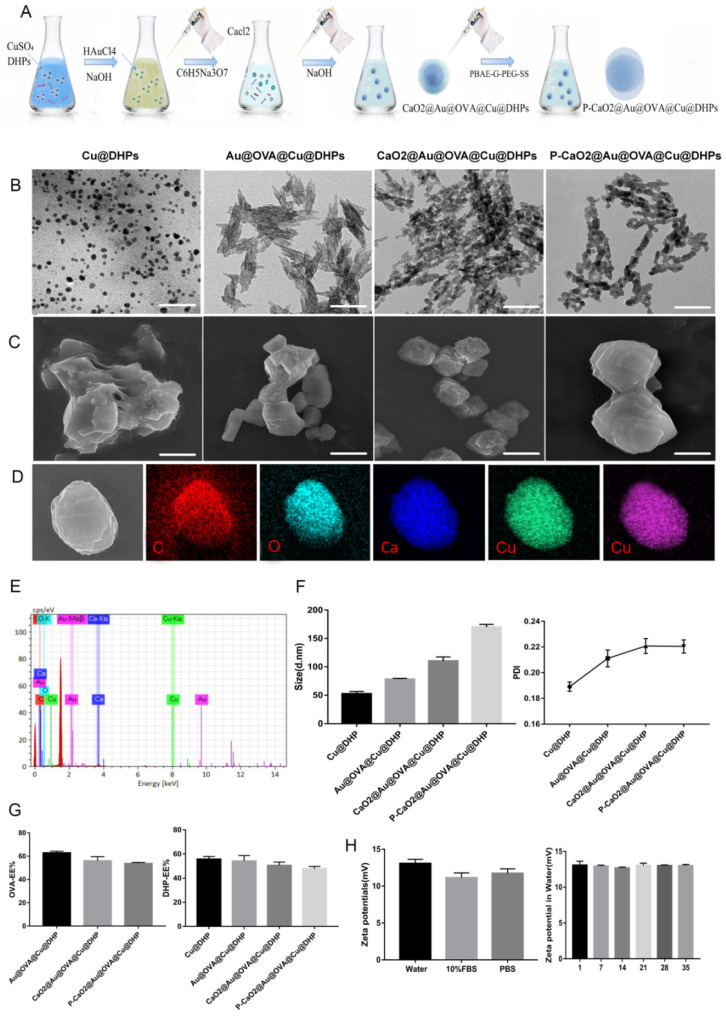
(**A**): An overview of the P-CaO_2_-Au@OVA@Cu@DHPs synthesis process. (**B**,**C**): SEM (**B**) and TEM (**C**) were used to visualize Cu@DHPs, Au@OVA@Cu@DHPs, CaO_2_-Au@OVA@Cu@DHPs, and P-CaO_2_-Au@OVA@Cu@DHPs NPs. (**D**,**E**): Elemental mapping results for P-CaO_2_-Au@OVA@Cu@DHPs NPs. (**F**): The size and PDI of P-CaO_2_-Au@OVA@Cu@DHPs NPs in an aqueous solution were assessed via DLS. (**G**): DHPs-EE (%) and OVA-EE (%) values were calculated for P-CaO_2_-Au@OVA@Cu@DHPs NPs. (**H**): ζ-potential values were measured for P-CaO_2_-Au@OVA@Cu@DHPs NPs in water, 10% FBS, and PBS, or were measured from days 1–35. Data are means ± SEM, n = 3.

The stimuli-responsive NPs developed in this study were designed to simultaneously encapsulate OVA and DHPs to provoke a robust immune response (Scheme 1 and Figure 2A). These NPs were composed of an outer hydrophilic poly(ε-caprolactone)-SS-poly(ethylene glycol)-poly(ε-caprolactone) shell, with a biodegradable hydrophobic core composed of PBAE-G-SS-PEG capable of encapsulating DHPs and OVA. Polysaccharides can be rapidly released from these NPs through the hydrophobic-to-hydrophilic conversion. The prepared polymer in the hydrophobic core can encapsulate the polysaccharide macromolecule and OVA. The prepared P-CaO_2_-Au@OVA@Cu@DHPs NPs were visualized via SEM and TEM as well-dispersed spherical particles with an average diameter of ~150 nm (Figure 2B,C). SEM further demonstrated that DHPs and OVA were encapsulated, yielding somewhat cube-like particles with a slightly rough texture. Following surface modification, these P-CaO_2_-Au@OVA@Cu@DHPs particles were spherical and still exhibited a slightly rough texture. TEM and SEM also highlighted the sheet-like morphological characteristics of these P-CaO_2_-Au@OVA@Cu@DHPs NPs. Elemental mapping of an individual P-CaO_2_-Au@OVA@Cu@DHPs particle revealed high levels of C (red) and O (blue) surrounding the Au (purple) core, consistent with uniformly distributed P-CaO_2_-Au@OVA@Cu@DHPs. Energy dispersive X-ray spectroscopy (EDS) revealed that these P-CaO_2_-Au@OVA@Cu@DHPs NPs were primarily composed of C, O, Ca, Au, and Cu, with visible Al signals being attributable to the Al mesh (Figure 2D,E).

XRS and XRD revealed that these P-CaO_2_-Au@OVA@Cu@DHPs NPs were primarily composed of C, O, Ca, Au, and Cu (Figures S1 and S2). In addition, FTIR revealed the successful construction of nanoparticles (Figure S3). P-CaO_2_-Au@OVA@Cu@DHPs NPs were slightly larger than CaO_2_-Au@OVA@Cu@DHPs particles, with an average diameter of 157.2 ± 2.21 nm. These samples exhibited PDI values below 0.3, consistent with a narrow size distribution (Figure 2F). Respective DHP encapsulation rates in Cu@DHPs, Au@OVA@Cu@DHPs, CaO_2_-Au@OVA@Cu@DHPs, and P-CaO_2_-Au@OVA@Cu@DHPs NPs were 55.7 ± 1.31%, 54.17 ± 2.60%, 50.53 ± 1.59%, and 47.8 ± 1.11% (Figure 2G), while the respective OVA encapsulation rates in Au@OVA@Cu@DHPs, CaO_2_-Au@OVA@Cu@DHPs, and P-CaO_2_-Au@OVA@Cu@DHPs NPs were 62.83 ± 0.81%, 56.03 ± 2.09%, and 53.73 ± 0.52%. P-CaO_2_-Au@OVA@Cu@DHPs NP stability was assessed based on ζ-potential values, with positively charged ζ-potentials for these NPs being measured in water (11.90 ± 0.71 mV), 10% FBS (12.30 ± 1.45 mV), and PBS (11.73 ± 1.11 mV) (Figure 2H). These ζ-potentials remained unchanged from days 1–35 (*p* > 0.05). Together, these findings confirmed the successful synthesis of P-CaO_2_-Au@OVA@Cu@DHPs NPs. In addition, we evaluated the optimal micelle concentration of P-CaO_2_-Au@OVA@Cu@DHPs, and the results are shown in Figure S4

### 3.3. Nanoparticle Stability Analyses

**Figure 3:**
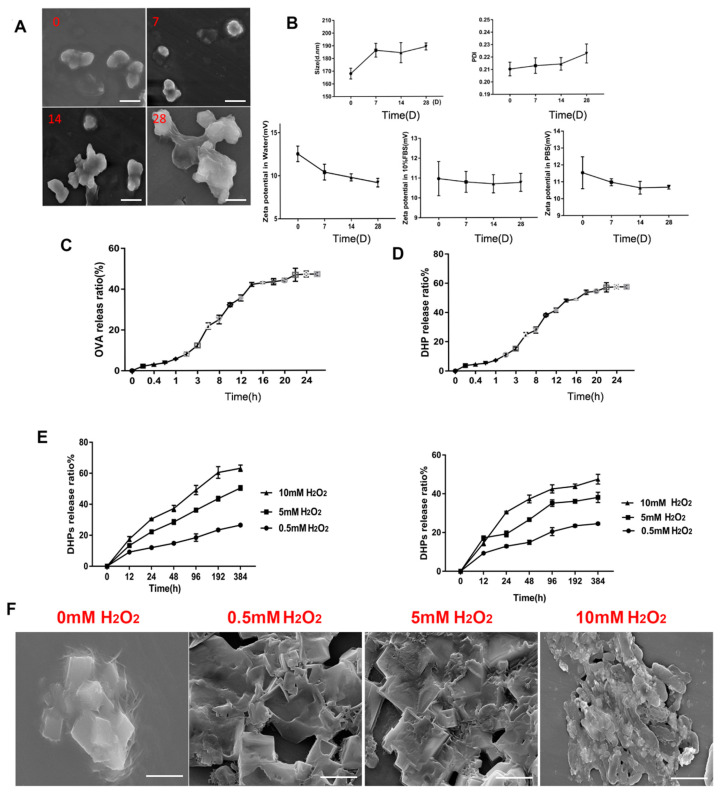
Analyses of P-CaO_2_-Au@OVA@Cu@DHPs NP stability. P-CaO_2_-Au@OVA@Cu@DHPs NP solutions were stored at 4 °C. (**A**): SEM was used to image particles on days 0, 7, 14, and 28. (**B**): NP size, PDI, and zeta potential were assessed on day 28. (**C**,**D**): The OVA (**C**) and DHP (**D**) release ratios (%) were measured over time from P-CaO_2_-Au@OVA@Cu@DHPs NPs. (**E**): The cumulative release of OVA and DHPs from NPs when incubated in PBS containing 0, 0.5, 5, or 10 mM H_2_O_2_ at 37 °C. (**F**): SEM images of P-CaO_2_-Au@OVA@Cu@DHPs NPs treated with PBS containing the indicated H_2_O_2_ concentrations at 37 °C (Scar bar = 100 nm).

P-CaO_2_-Au@OVA@Cu@DHPs NP stability was next assessed using TEM to evaluate the morphological characteristics of these NPs over the course of a 4-week incubation at 4 °C, revealing no apparent morphological changes during this interval (Figure 3A). P-CaO_2_-Au@OVA@Cu@DHPs NP particles exhibited diameters that rose to 186.2 nm from 172.5 nm on day 28 (Figure 3B), and all PDI values on day 28 remained below 0.3, with no significant changes in these values, consistent with a homogeneous particle distribution. Zeta potential values for these NPs were similarly unchanged over this 28-day incubation.

### 3.4. Examination of the ROS- and pH-Responsive Characteristics of P-CaO_2_-Au@OVA@Cu@DHPs NPs

On day 1, OVA encapsulated within P-CaO_2_-Au@OVA@Cu@DHPs NPs was slowly released (cumulative release: 5.73 ± 0.06%) (Figure 3C), with relatively rapid release over days 1–16 for a cumulative release of 42.97 ± 0.20%. From days 16–36, this cumulative release rate rose to 47.15 ± 0.56%, potentially owing to the breakdown of the PBAE-G-SS-PEG polymer and structural damage to the CaO_2_ emulsion, leading to accelerated OVA release. These P-CaO_2_-Au@OVA@Cu@DHPs NPs thus exhibit robust controlled-release characteristics. In vitro DHP release from these P-CaO_2_-Au@OVA@Cu@DHPs NPs was similarly assessed (Figure 3D), revealing a low release rate on day 1 that rose rapidly for a cumulative release of 57.25 ± 0.12% on day 36. PBAE has been reported to be highly responsive to changes in pH and ROS levels [34]. The OVA and DHP release rates from the prepared NPs in biofluid mimetic solutions were just 15% within 48 h, whereas when H_2_O_2_ was present, these release rates rose markedly, which is consistent with the ROS-responsive properties of these NPs (Figure 3E,F).

### 3.5. Assessment of the Cytotoxicity and Uptake of P-CaO_2_-Au@OVA@Cu@DHPs NPs When Used to Treat Macrophages

**Figure 4:**
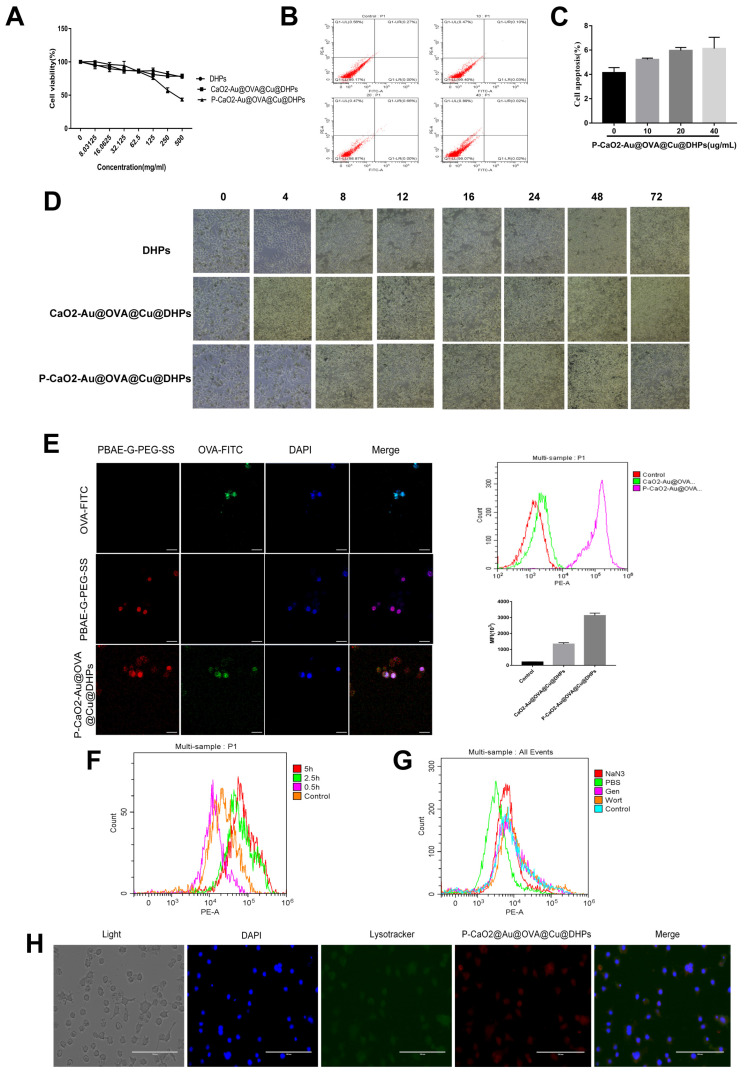
Examination of the cytotoxicity and uptake of DHPs and P-CaO_2_-Au@OVA@Cu@DHPs NPs by macrophages. (**A**): Macrophage viability was quantified following incubation with DHPs or P-CaO_2_-Au@OVA@Cu@DHPs NPs for 48 h. (**B**): Flow cytometry was used to detect cytotoxicity in macrophages. (**C**): macrophage morphology was assessed following DHP or P-CaO_2_-Au@OVA@Cu@DHPs NP treatment for 24 or 48 h. (**D**): Macrophages treated with OVA-FITC, PBAE-G-PEG-SS, and P-CaO_2_-Au@OVA@Cu@DHPs NPs were imaged via CLSM. (**E**): Flow cytometry was used to assess macrophages cultured with P-CaO_2_-Au@OVA@Cu@DHPs NPs and CaO_2_-Au@OVA@Cu@DHPs NPs. (**F**): Flow cytometry was used to assess macrophages cultured for 0.5, 2.5, or 5 h in the presence of P-CaO_2_-Au@OVA@Cu@DHPs NPs. (**G**): Colocalization assay. (**H**): The inhibition of uptake was assessed in macrophages treated with various endocytic inhibitors.

The cytotoxicity of both free DHPs and P-CaO_2_-Au@OVA@Cu@DHPs NPs was next examined through flow cytometry and MTT assays. Macrophages were treated for 48 h with a range of DHP or P-CaO_2_-Au@OVA@Cu@DHPs NP concentrations, revealing that neither of these preparations was toxic at a dose of 30 μg/mL (Figure 4A), such that this dose was used in subsequent experiments. The ability of macrophages to efficiently take up antigens is required for antigen presentation and immune response induction [34]. Macrophages serve as first-line innate immune defenders against pathogen invasion. Macrophage antigen uptake was assessed by confocal laser scanning microscopy (CLSM) for cells incubated with OVA-FITC, PBAE-G-PEG-SS, and P-CaO_2_-Au@OVA@Cu@DHPs NPs, revealing higher levels of OVA-FITC internalization in the P-CaO_2_-Au@OVA@Cu@DHPs NP group as compared to cells incubated with OVA-FITC alone (Figure 4B). P-CaO_2_-Au@OVA@Cu@DHPs NPs were also internalized more readily than CaO_2_-Au@OVA@Cu@DHPs NPs in this assay system (Figure 4B). The endocytic uptake of P-CaO_2_-Au@OVA@Cu@DHPs NPs was further examined by treating these macrophages with endocytosis inhibitors including NaN_3_ (120 mM), wortmannin (Wort, 0.2 μM), and genistein (Gen, 200 μM). Flow cytometry revealed a reduction in fluorescent signal within cells treated with these inhibitors, with NaN_3_ having a particularly suppressive effect on P-CaO_2_-Au@OVA@Cu@DHPs NP internalization, suggesting that P-CaO_2_-Au@OVA@Cu@DHPs NPs can be internalized via endocytosis. These findings support the ability of P-CaO_2_-Au@OVA@Cu@DHPs NPs to efficiently promote macrophage-mediated antigen uptake.

### 3.6. P-CaO_2_-Au@OVA@Cu@DHPs NPs Influence Costimulatory Marker Expression, Cytokine Secretion, and cGAS–STING Signaling In Vitro

**Figure 5:**
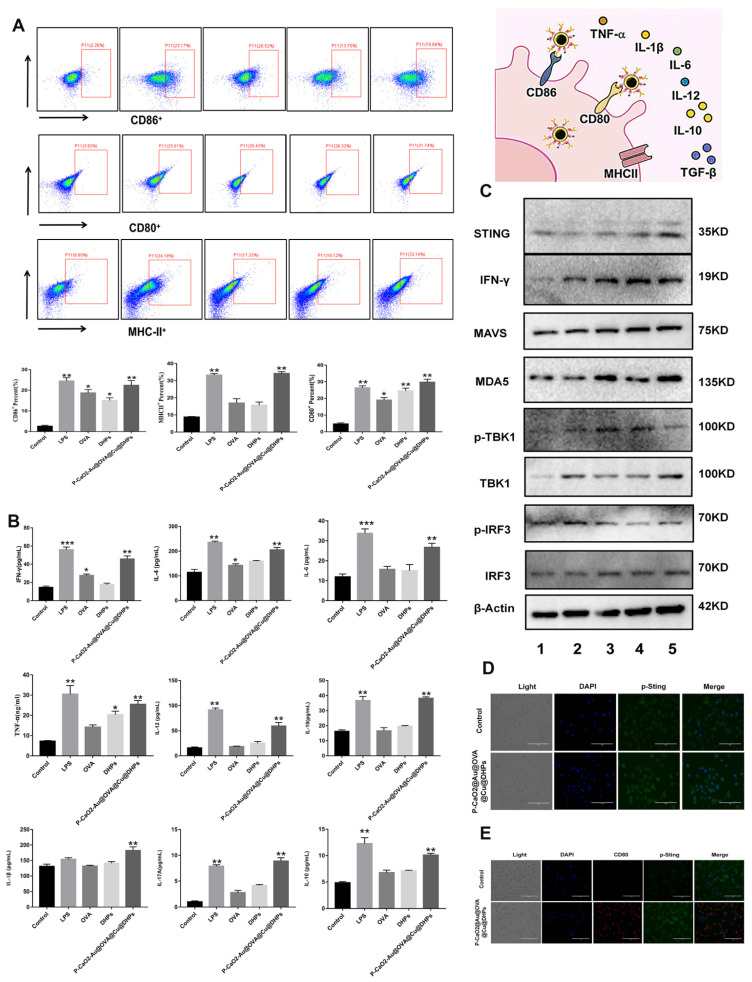
Analysis of the in vitro NP-induced changes in activation marker expression, cytokine production, and cGAS–STING pathway activity. (**A**): Flow cytometry was used to assess MHCII, CD80, and CD86 expression on macrophages. (**B**): Macrophage-derived IL-2, IL-4, IL-1β, IL-6, IFN-γ, and TNF-α levels were quantified. (**C**): Western immunoblotting was used to assess the expression of cGAS–STING pathway proteins in macrophages treated for 24 h under the indicated conditions, with β-actin as a loading control (1—Control, 2—LPS, 3—OVA, 4—DHPs, 5—P-CaO_2_-Au@OVA@Cu@DHPs). (**D**): Representative CLSM images showing p-STING levels in P-CaO_2_-Au@OVA@Cu@DHPs NP-treated macrophages. (**E**): Representative CLSM images showing CD80 and p-STING protein levels in CT26 cells (blue, DAPI; green, Alexa Fluor 488 Phalloidin). Note: Significant difference compared with control group, * *p* < 0.05, ** *p* < 0.01, *** *p* < 0.001.

After phagocytosing pathogen- or vaccine-derived antigens, activated macrophages can present these peptides to T cells to induce adaptive immunity [35], contributing to the establishment of both cellular and humoral immune responses [36]. After antigen uptake, macrophages generally express high levels of costimulatory proteins [37], with increased MHCII, CD80, and CD86 expression on these cells being conducive to the activation of T cells. The impact of P-CaO_2_-Au@OVA@Cu@DHPs NP treatment on macrophages was assessed by using flow cytometry to detect cell surface MHCII, CD80, and CD86 levels on these cells, revealing that all three were significantly upregulated following P-CaO_2_-Au@OVA@Cu@DHPs NP treatment relative to the PBS control group (*p* < 0.05), with no significant difference relative to the LPS positive control group (*p* > 0.05) (Figure 5A). Cytokine secretion from macrophages and other immune cells is also vital for the appropriate induction and regulation of immune responses [38]. Accordingly, ELISAs were used to measure IL-2, IL-4, IL-1β, IL-6, IFN-γ, and TNF-α levels in supernatants harvested from P-CaO_2_-Au@OVA@Cu@DHPs NP-treated macrophages, revealing that relative to free DHPs, OVA, or control treatment, these NPs induced higher levels of secretion for all of these analyzed cytokines (*p* < 0.05 and *p* < 0.01), with no significant differences between the P-CaO_2_-Au@OVA@Cu@DHPs NP and LPS groups (Figure 5B).

Innate immunity served as the first line of defense against pathogenic incursion [38], with the recognition of pathogen- and damage-associated molecular patterns rapidly stimulating these innate immune responses mediated by pattern recognition receptors (PRRs). The cyclic guanosine monophosphate (GMP)-adenosine monophosphate (AMP) synthase (cGAS)-stimulator of interferon genes (STING) pathway is an important DNA-sensing PRR signaling axis that protects against an array of pathogens. Macrophages were treated for 24 h with LPS, OVA, DHPs, or P-CaO_2_-Au@OVA@Cu@DHPs NPs, after which cGAS–STING pathway proteins were detected through Western immunoblotting. This approach revealed that p-TBK1, TBK1, p-IRF3, IRF3, MDA5, STING, and p-STING were all upregulated in response to P-CaO_2_-Au@OVA@Cu@DHPs NP treatment (Figure 5C). Immunofluorescent staining and CLSM imaging additionally confirmed a pronounced increase in p-STING levels in P-CaO_2_-Au@OVA@Cu@DHPs NP-stimulated macrophages relative to control cells (Figure 5D). Higher levels of both CD80 and p-STING were also evident in cells treated with P-CaO_2_-Au@OVA@Cu@DHPs NPs relative to control cells.

### 3.7. P-CaO_2_-Au@OVA@Cu@DHPs NPs Induce Robust T Cell-Mediated Immune Responses

**Figure 6:**
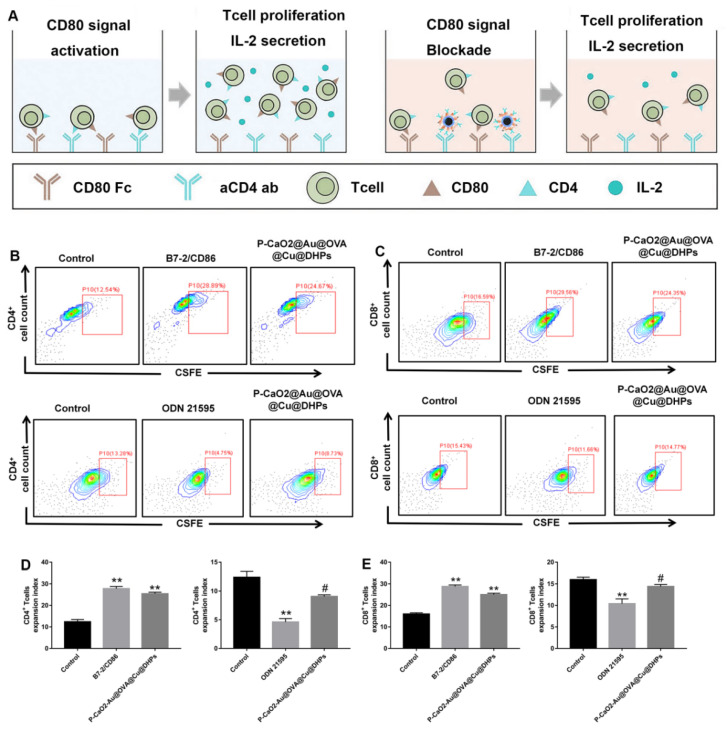
Evaluation of the ability of P-CaO_2_-Au@OVA@Cu@DHPs to activate T cells. T cells were added to plates pre-coated with anti-CD3 and a CD80-Fc fusion protein, after which P-CaO_2_-Au@OVA@Cu@DHPs NPs, the CD80-activating agent B7-2, or the CD80-inhibiting agent ODN 21595 were added. (**A**): An overview of the utilized experimental approach. (**B**,**C**): Representative flow cytometry plots assessing CSFE dilution in CD4+ (**B**) and CD8+ (**C**) T cells in the indicated treatment groups. (**D**,**E**): Flow cytometry was used to establish the expansion indices for CD4+ (**D**) and CD8+ (**E**) T cells. # *p* < 0.01 (P-CaO_2_-Au@OVA@Cu@DHPs group compared with the B7-2/CD86 group), ** *p* < 0.01 (compared with the control group). Note: Significant difference compared with control group, ** *p* < 0.01.

Cellular immunity coordinated by CD4^+^ and CD8^+^ T cells is essential for robust protection against many pathogens [38]. The activation of these relies on multiple signals. After recognizing and processing antigens, APCs can generate MHC-peptide complexes that display antigens to immune cells such that they can bind to cognate T cell receptors. Co-stimulatory molecules on the surface of these APCs can also interact with cognate cell surface receptors found on T cells to strengthen the interaction, stimulating appropriate activation. Splenic T cells were used to assess the ability of P-CaO_2_-Au@OVA@Cu@DHPs NPs to induce the activation of cellular immunity, with both T cell expansion and IL-2 secretion being measured as readouts for T cell activation. Anti-CD3- and CD80-Fc-coated plates were used to mimic the interactions between T cells and APCs (Figure 6A). As shown in Figure 6B, B7-2 promotes the production of mature APC subpopulations and promotes APC function and survival. B7 protein is also involved in innate immune responses by activating the NF-κB signaling pathway in macrophages. P-CaO_2_-Au@OVA@Cu@DHPs NPs have the same immune-activating effect compared with B7-2, which could stimulate the expression level of CD4^+^ and CD8^+^ (Figure 6B–E). Interestingly, ODN 21,595 as a CD86 inhibitor could inhibit the expression level of CD4^+^ and CD8^+^. Above all, P-CaO_2_-Au@OVA@Cu@DHPs NPs effectively induced T cell expansion.

### 3.8. IP-CaO_2_-Au@OVA@Cu@DHPs/mRNA Vaccine Complexes Induce Robust In Vivo Immunity

**Figure 7:**
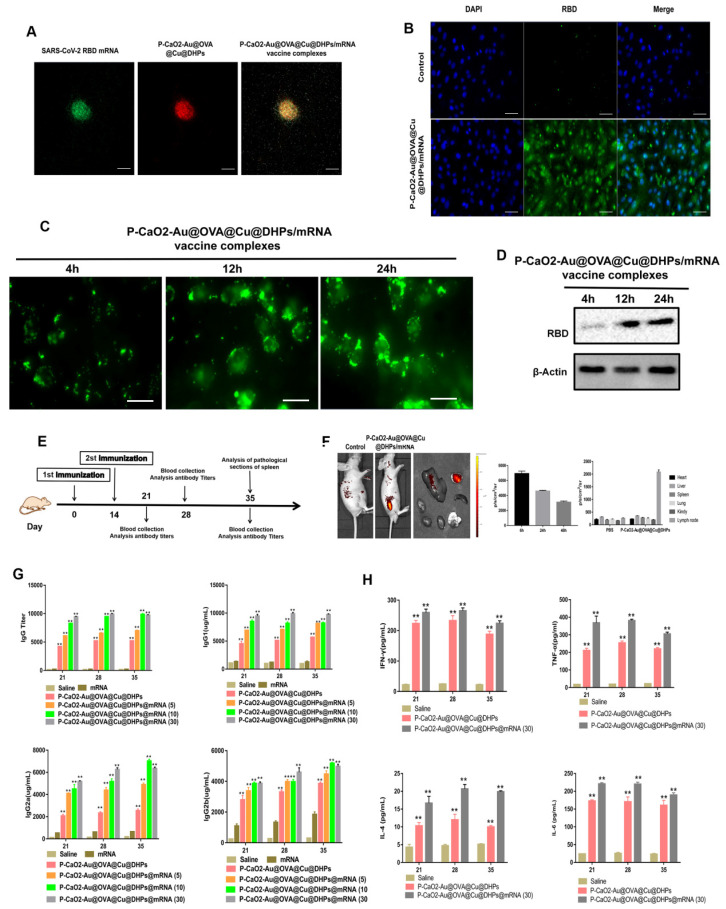
Analyses of P-CaO_2_-Au@OVA@Cu@DHPs/mRNA vaccine complex-induced immune responses. (**A**): Representative CLSM images showing the prepared P-CaO_2_-Au@OVA@Cu@DHPs/mRNA vaccine complexes. (**B**,**C**): At 24 h following P-CaO_2_-Au@OVA@Cu@DHPs/mRNA complex transfection, HEK-293T cells were examined by fluorescence microscopy and flow cytometry to detect the eGFP signal. (**D**): Western immunoblotting was used to detect RBD protein levels following HEK-293T cell transfection with the prepared P-CaO_2_-Au@OVA@Cu@DHPs/mRNA vaccine complex. (**E**): Schematic overview of the in vivo immunization strategy. Mice were immunized on day 0 with a booster dose on day 14 and were euthanized and serum samples were collected on days 21, 28, 35, and 42. Spleens were collected on day 28 for histological examination. Control mice were instead administered PBS. (**F**): Bioluminescent imaging was used to gauge the efficacy of in vivo vaccine delivery. (**G**): RBD-specific IgG titers were assessed over time following vaccination, as were IgG1, IgG2a, and IgG2b titers. (**H**): ELISAs were used to detect Th1 (IFN-γ, TNF-α) and Th2 (IL-4, IL-6) cytokines in the serum of immunized mice. Data are means ± SEM, n = 4. Note: Significant difference compared with control group, ** *p* < 0.01.

CLSM was used to image the prepared P-CaO_2_-Au@OVA@Cu@DHPs/mRNA vaccine complexes (Figure 7A), revealing the presence of the green fluorescent SARS-CoV-2 RBD mRNA and the red fluorescence of P-CaO_2_-Au@OVA@Cu@DHPs. These P-CaO_2_-Au@OVA@Cu@DHPs/mRNA complexes were then used to transfect HEK-293T cells, after which RBD-eGFP expression was assessed as a marker for transfection efficiency (Figure 7B,C). Western immunoblotting was further used to confirm that RBD was successfully expressed by HEK-293T cells transfected with this P-CaO_2_-Au@OVA@Cu@DHPs/mRNA vaccine (Figure 7D).

To better explore the utility of P-CaO_2_-Au@OVA@Cu@DHPs NPs as an adjuvant for mRNA vaccines in vivo, mice were intramuscularly immunized with a range of P-CaO_2_-Au@OVA@Cu@DHPs/mRNA vaccine complex doses (5 μg, 10 μg, or 30 μg/mouse, n = 5/dose) (Figure 7E). A clearly fluorescent signal was evident after dosing, confirming that the injection and protein expression were successful (Figure 7F). RBD-specific IgG was further detected via ELISA as a measure of humoral immunity (Figure 7G), with pronounced antibody titers being evident on day 21 after the first immunization with P-CaO_2_-Au@OVA@Cu@DHPs/mRNA complexes. After booster immunization, these antibody levels rose rapidly. A dose-dependent relationship was observed between P-CaO_2_-Au@OVA@Cu@DHPs/mRNA dose and antibody concentrations, with mean endpoint titers >10^5^ in the 30 μg group, with these levels being, respectively, 1.5- and 5.2-fold higher than in the 10 μg and 5 μg groups. The 30 μg dose was selected for all subsequent immunizations, and IgG1, IgG2a, and IgG2b titers were measured in immunized mice (Figure 7G). Cytokines were also measured via ELISA on days 21, 28, and 35 after initial immunization. This analysis revealed that P-CaO_2_-Au@OVA@Cu@DHPs/mRNA induced significantly higher Th1 cytokine (IFN-γ and TNF-α) levels relative to other treatment conditions (*p* < 0.05), with TNF-α serving as an effective inducer of Th1 responses. Similar trends in IL-4 and IL-6 secretion were also observed, with P-CaO_2_-Au@OVA@Cu@DHPs/mRNA inducing higher levels than other treatments (*p* < 0.05) (Figure 7H). This suggests that the P-CaO_2_-Au@OVA@Cu@DHPs/mRNA vaccine can effectively induce Th1 and Th2 cytokines, consistent with the observed antibody titers.

### 3.9. P-CaO_2_-Au@OVA@Cu@DHPs/mRNA Vaccine Complexes Induce Immune Cell Activation via the cGAS–STING and MDA5–IFN-α Signaling Pathways

P-CaO_2_-Au@OVA@Cu@DHPs and mRNA were complexed by electrostatic interaction. The ability of P-CaO_2_-Au@OVA@Cu@DHPs to bind mRNA was verified by gel retardation assay. The P-CaO_2_-Au@OVA@Cu@DHPs/mRNA complexes at different N/P ratios (1, 2, 4, 8, 16, 32) were applied, and mRNA was efficiently retarded by P-CaO_2_-Au@OVA@Cu@DHPs at an N/P ratio of 4 (Figure S5). To evaluate the transfection efficiency of PVES/mRNA complexes, eGFP mRNA was used as the reporter gene. HEK-293T cells were transfected with P-CaO_2_-Au@OVA@Cu@DHPs/mRNA complexes at different N/P ratios (Figure S6). The results indicated that the transfection efficiency was greatly enhanced with the increase of N/P ratios and reached a plateau at N/P = 32. As an RNA virus, the innate immune recognition of SARS-CoV-2 is primarily achieved through the RIG-I-like receptors [39], but a growing number of studies have also established the importance of the cGAS DNA sensor in the restriction of RNA virus infections, with many RNA viral proteins serving to suppress cGAS–STING signaling activity [40].

**Figure 8:**
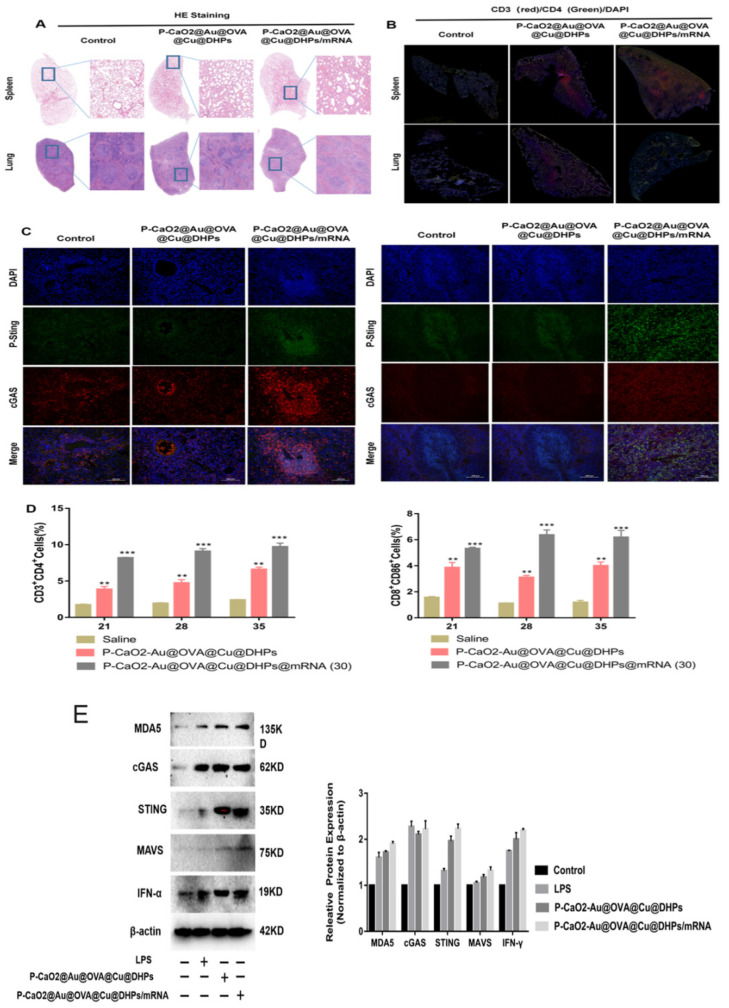
The P-CaO_2_-Au@OVA@Cu@DHPs/mRNA complex induces in vivo immune responses via the cGAS–STING pathway. (**A**): Spleen and lung tissue samples from mice were subjected to H&E staining on day 35 following booster immunization. (**B**,**C**): Immunofluorescent staining of lung and spleen tissue samples from mice in the indicated groups was assessed to detect CD3 and CD4 (**B**) or p-STING and cGAS (**C**). (**D**): The frequencies of CD3+ and CD8+ T cells (gated on CD3+ T cells) were assessed in the spleen. (**E**): The indicated cGAS–STING and MDA–IFN-α-related proteins were assessed in the spleens of P-CaO_2_-Au@OVA@Cu@DHPs/mRNA-immunized mice via Western immunoblotting. Data are means ± SEM, n = 4. Note:Significant difference compared with control group, ** *p* < 0.01, *** *p* < 0.001.

Moreover, cGAS–STING pathway activation can enhance dendritic cell (DC)-mediated cross-presentation through type I interferon (IFN) production, thereby specifically activating robust antiviral CD8^+^ T cell responses [41]. The SARS-CoV-2 spike protein is also capable of stimulating innate immunity through the activation of Toll-like receptors 2 and 4 (TLR2 and TLR4) [42]. The TLR5 pathway also serves an important role in the microbiota-mediated regulation of immune responses to non-adjuvant-containing vaccines such as the seasonal flu vaccine. mRNA, P-CaO_2_-Au@OVA@Cu@DHPs, and P-CaO_2_-Au@OVA@Cu@DHPs/mRNA induced no obvious pathologic changes (Figure 8A), which were similar to the results with the saline control group. Immunofluorescent staining further confirmed marked increases in CD3 and CD4 staining intensity in the lungs and spleens of these P-CaO_2_-Au@OVA@Cu@DHPs/mRNA complex-immunized mice, consistent with the induction of a more robust T cell response (Figure 8B).

The COVID-19 BNT162b2 mRNA vaccine (Pfizer and BioNTech) can specifically stimulate a robust CD8^+^ T cell-mediated immune response through the MDA5–IFN-α signaling pathway. To evaluate the in vivo pathways through which P-CaO_2_-Au@OVA@Cu@DHPs/mRNA complexes can induce immunity in mice, samples of lung and spleen tissue were stained to detect p-STING and cGAS prior to CLSM imaging. Stronger staining for both of these proteins was evident in the lungs and spleens of P-CaO_2_-Au@OVA@Cu@DHPs/mRNA-immunized mice, consistent with the enhancement of cGAS–STING protein expression and activity in these tissue compartments (Figure 8C,D). P-CaO_2_-Au@OVA@Cu@DHPs/mRNA immunization also increased the splenic MDA5, cGAS, STING, and IFN-*γ* protein levels in mice relative to the control and LPS treatment groups (Figure 8E,F). Overall, these results support the ability of P-CaO_2_-Au@OVA@Cu@DHPs/mRNA vaccine complexes to stimulate cellular immunity through the cGAS–STING and MDA5–IFN-α signaling pathways.

### 3.10. Evaluation of the In Vivo Biosafety of P-CaO_2_-Au@OVA@Cu@DHPs NPs

**Figure 9:**
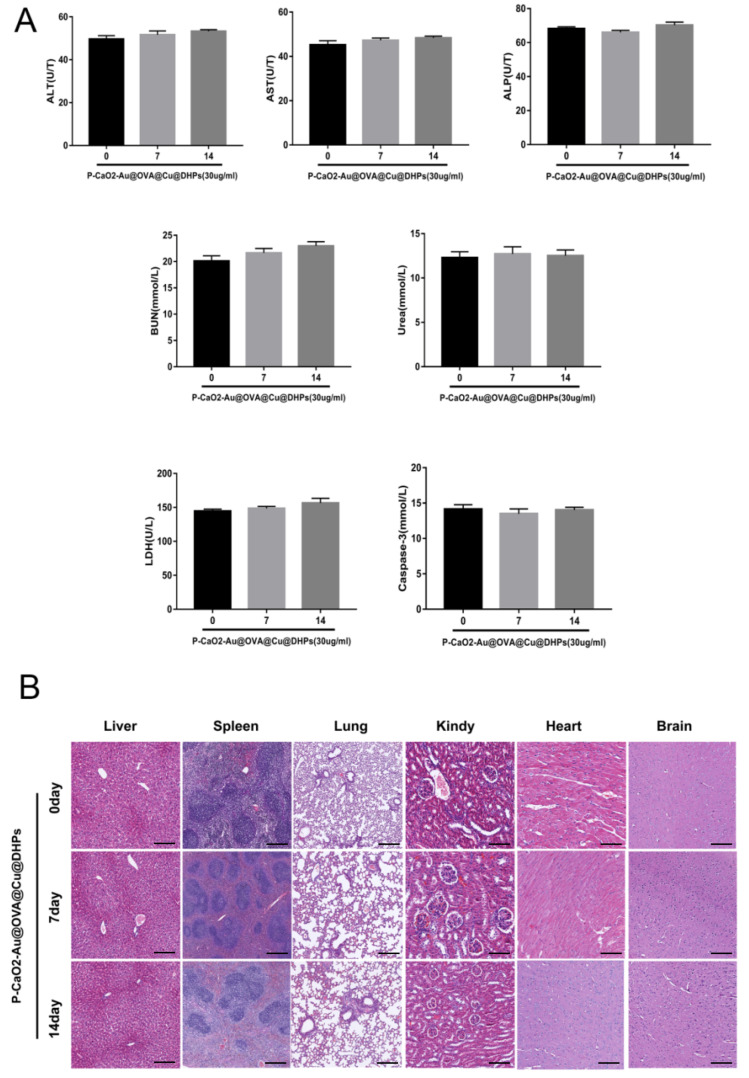
Evaluation of the in vivo biosafety of P-CaO_2_-Au@OVA@Cu@DHPs NPs. (**A**): Serum ALT, AST, ALP, LDH, and BUN levels were analyzed in mice administered P-CaO_2_-Au@OVA@Cu@DHPs NPs. Data are means ± SEM, n = 3. (**B**): Samples of spleen, lung, heart, kidney, and liver tissue from mice administered P-CaO_2_-Au@OVA@Cu@DHPs were subjected to H&E staining. Scale bar: 40 μm.

To explore the biosafety of the prepared P-CaO_2_-Au@OVA@Cu@DHPs NPs in vivo when used as a vaccine and adjuvant delivery platform, major organs including the spleen, liver, heart, kidneys, and lungs of P-CaO_2_-Au@OVA@Cu@DHPs NP-treated mice were examined via H&E staining. This approach revealed no apparent histological abnormalities in these mice relative to control animals treated with PBS, consistent with the absence of any apparent inflammation or damage (Figure 9B). Serum biochemistry assays were also used to assess markers of liver function (ALT, AST, and ALP), myocardial function (LDH), and renal function (BUN), as increases in any of these markers would be indicative of impaired liver, renal [42], or myocardial function. No changes in any of these biochemical indices were evident in P-CaO_2_-Au@OVA@Cu@DHPs NP-treated mice (Figure 9A), consistent with the safety of utilizing these NPs as a vaccine adjuvant and delivery platform.

## 4. Discussion

COVID-19 is a complex and devastating disease that causes both respiratory and systemic symptoms, suggesting that SARS-CoV-2 is likely to provoke robust systemic immunity and a broadly dysregulated immune response. Vaccination is vital in the prevention of viral diseases as it can induce durable antigen-specific immune responses. While the COVID-19 pandemic spurred vaccine development with unprecedented rapidity, there remains a persistent need for new broadly protective SARS-CoV-2 vaccines owing to antigenic drift and the emergence of novel viral variants. The BNT162b2 vaccine developed by Pfizer and BioNTech is the first mRNA vaccine to have ever received US Food and Drug Administration (FDA) approval. While effective, these mRNA vaccines are thought to be less immunogenic and less secure relative to more traditional vaccines [43]. There is also a need for the development of more effective mRNA delivery systems, since mRNA molecules are large and negatively charged such that they cannot readily traverse the plasma membrane. Moreover, mRNA exhibits poor stability and is readily degraded [44].

The advent of novel mRNA delivery systems and more potent adjuvants for these vaccines thus has the potential to stimulate more robust cellular and humoral immune responses, underscoring a major unmet clinical need. A range of approaches to mRNA delivery, including liposomes, lipoplexes, and lipid NPs, have been designed. Polyethyleneimine (PEI) has been shown to exhibit good buffering capacity and a cationic charge that is conducive to nucleic acid complexing and release within the endolysosomal compartment through a proton-sponge effect [45]. Low-molecular-weight PEI is commonly applied as a less toxic approach to mRNA delivery [46]. NP-based adjuvant delivery approaches have also become increasingly popular as they can shield antigens and drugs from degradation while enabling their internalization by APCs, thereby enhancing immune response induction. Nanoparticles exhibit a high specific surface area, can be readily targeted, and are highly biocompatible such that they are well suited to biomedical applications [47], including as drug delivery [47] and gene therapy vectors. In the present study, nanoparticles with a hydrophilic modified chimeric PBAE shell layer were used to enhance immunity through adjuvant activity while also delivering an mRNA payload.

For the present study, bimetallic NPs were prepared and loaded with OVA and polysaccharides as reported previously [48]. PEI and DSPE-PEG2000-SS are water-soluble polymers while PBAE is hydrophobic, and PEI and DSPE-PEG2000-SS can be covalently linked to PBAEs to generate amphiphilic co-polymers capable of self-assembling into stable micelles [30]. The hydrophilic and chimeric modified PBAEs shell of these P-CaO_2_-Au@OVA@Cu@DHPs NPs was thus generated using a one-pot approach in this study, yielding homogeneous spherical NPs as visualized via SEM and TEM with a low PDI below 0.3. The prepared CaO_2_-Au@OVA@Cu@DHPs and P-CaO_2_-Au@OVA@Cu@DHPs NPs exhibited respective diameters of 115 nm and 167 nm, with this size being conducive to the formation of P-CaO_2_-Au@OVA@Cu@DHPs/mRNA complexes via electrostatic interactions, enabling endocytic uptake of these particles while avoiding rapid reticuloendothelial system (RES)-mediated clearance in vivo.

The ability of P-CaO_2_-Au@OVA@Cu@DHPs NPs to induce antibody responses is essential for their effective utility as a vaccine adjuvant platform. Adjuvants can aid in the production of a wide range of diverse antibody responses conducive to robust protective immunity [49]. In this study, P-CaO_2_-Au@OVA@Cu@DHPs NPs effectively increased antigen-specific IgG levels in mice following immunization, consistent with excellent vaccine and adjuvant activity. These NPs are also stable and positively charged, enabling the absorption of negatively charged mRNA through electrostatic adsorption. All three tested P-CaO_2_-Au@OVA@Cu@DHPs/mRNA vaccine complex doses effectively induced serum IgG specific for SARS-CoV-2 RBD, confirming the dose-dependent effects of P-CaO_2_-Au@OVA@Cu@DHPs vaccine delivery. This mRNA vaccine candidate thus holds great promise as an efficient vaccine delivery vehicle.

Both macrophages and DCs function as professional APCs that are essential for the stimulation of potent immunologic responses. These APCs are capable of internalizing, processing, and presenting antigens to B and T lymphocytes to provoke their activation and maturation. Macrophage antigen uptake is also critical for the generation of effective immunity. When activated, macrophages upregulate MHCII and costimulatory molecules, including CD86, to activate T cells [19]. In this study, the ability of P-CaO_2_-Au@OVA@Cu@DHPs NPs to activate and be internalized by macrophages was assessed. Cellular immune responses are vital to vaccine-induced immunity, with macrophages, B cells, and T cells all coordinating this response through the production of cytokines. These include TNF-α, which is a proinflammatory cytokine that bolsters immune cell activation and migration, as well as IL-6, which can induce the production of the Th2 cytokine IL-4. In this study, P-CaO_2_-Au@OVA@Cu@DHPs NPs enhanced macrophage-mediated TNF-α and IL-6 secretion, while P-CaO_2_-Au@OVA@Cu@DHPs/mRNA complexes significantly increased the circulating IL-4 and IL-6 levels in mice. IL-4 can promote B cell proliferation and maturation. IFN-γ is also important for the modulation of cellular immune responses, coordinating a range of antiviral functions.

SARS-CoV-2-specific mRNA vaccines have been reported to activate a range of PRR signaling mechanisms, including the TLR4, TLR7, and cGAS–STING pathways, provoking a robust innate immune response. cGAS is a cytoplasmic DNA receptor that can be activated by double-stranded DNA. Once activated, cGAS catalyzes the cyclization of cytoplasmic adenosine and guanosine monophosphate into cyclic guanosine monophosphate–adenosine monophosphate (cGAMP). Subsequently, cGAMP binds to the V-shaped binding pocket of the STING dimer, causing conformational changes, aggregation, and activation of STING. The activated STING recruits and activates the kinase TBK1, which phosphorylates interferon regulatory factor 3 (IRF-3) to regulate the expression of downstream genes. At the same time, the activation of STING also recruits IKK kinase, releases nuclear factor κB (NF-κB), and induces the expression of interferons (IFNs) and pro-inflammatory cytokines (such as tumor necrosis factor (TNF), interleukin 1 (IL-1), and interleukin 6 (IL-6)). Type I interferons have multiple immune-stimulating functions and can promote the maturation, migration, and activation of immune cells such as dendritic cells (DCs), T cells, and natural killer cells (NK). In this report, the ability of P-CaO_2_-Au@OVA@Cu@DHPs NPs to activate innate immunity was assessed, revealing the ability of these NPs to induce immunity via the cGAS–STING and MDA5–IFN-α signaling pathways. Further preclinical assessment of the biosafety profile and clearance of P-CaO_2_-Au@OVA@Cu@DHPs NPs is warranted given that this is an emerging biomaterial, but prior studies have shown that PBAGs-PEG-SS polymers can be readily broken down by human peroxidase enzyme activity. In addition, Au-Cu sheets administered intravenously can reportedly be excreted in the organ and cleared from internal organs. Consistently, P-CaO_2_-Au@OVA@Cu@DHPs NPs did not induce apparent toxicity in treated mice in the present analysis, although more detailed clinical evaluation will be required in the future.

In conclusion, we herein developed P-CaO_2_-Au@OVA@Cu@DHPs NPs as a novel mRNA vaccine delivery platform. These P-CaO_2_-Au@OVA@Cu@DHPs NPs were readily internalized by cells and induced immune activation in vitro. Moreover, these P-CaO_2_-Au@OVA@Cu@DHPs were able to induce potent cellular and humoral immune responses in vivo after intramuscular administration without any apparent toxicity, underscoring the promise of these NPs as a safe and effective mRNA vaccine delivery platform.

## Data Availability

All data are available from the corresponding author on reasonable request.

## Supplementary Materials

The following supporting information can be downloaded at: https://www.mdpi.com/article/10.3390/polym17192636/s1, Figure S1: XRS of P-CaO_2_-Au@OVA@Cu@DHPs; Figure S2: XRD of P-CaO_2_-Au@OVA@Cu@DHPs; Figure S3. FTIR of Cu@DHPs, Au@OVA@Cu@DHPs, CaO_2_-Au@OVA@Cu@DHPs and P-CaO_2_-Au@OVA@Cu@DHPs; Figure S4: CMC of P-CaO_2_-Au@OVA@Cu@DHPs; Figure S5: Gel retardation assays to detect condensation of mRNA into P-CaO_2_-Au@OVA@Cu@DHPs at different N/P ratios (left) and protection efficiency from RNase A degradation at N/P ratios of 16 and 32 (right); Figure S6: In vitro P-CaO_2_-Au@OVA@Cu@DHPs/mRNA complexes transfection and expression in HEK-293T cells. After 24 h transfection, expression of eGFP mRNA were analyzed by flow cytometry. eGFP expression efficiency was evaluated by the number of GFP positive cells measured by flow cytometry. Lipofectamine 3000, PEI 25 k and PEI 1.8 k were used as controls. Data were shown as mean ± SEM.

## Abbreviations

mRNA	Messenger RNA
SARS-CoV-2	Severe acute respiratory syndrome coronavirus 2
PBAE-G-B-SS	Poly(beta-amino ester)s
OVA	Ovalbumin
DHPs	Dendrobium polysaccharides
cDNA	Complementary DNA
shRNA	Short hairpin RNA
siRNA	Small interfering RNA
LOD	Limit of Detection
LOQ	Limit of Quantitation
RSD	Standard Deviation
DAPI	4′,6-Diamidino-2-phenylindole
BSA	Bovine albumin
IFN	Interferon
IL-6	Interleukin-6
TNF	Tumor necrosis factor
ANOVA	Analysis of Variance
