# Supplementary material for: Multifunctional Polymer-Modified P-CaO2@Au@OVA@Cu@DHPs Nanoparticles Enhance SARS-CoV-2 mRNA Vaccine-Induced Immunity via the cGAS–STING Signaling Pathway
Source: Polymers (Basel). 2025 Sep 30;17(19):2636. doi: 10.3390/polym17192636 (PMC12526564; doi:10.3390/polym17192636)
Supplement: Supplementary file 1 [file polymers-17-02636-s001.zip › polymers-3567500-supplementary.pdf]

Multifunctional polymer-modified P-CaO_2@Au@OVA@Cu@DHPs nanoparticles enhance SARS-CoV-2 mRNA vaccine-induced immunity via the cGAS-STING signaling pathway

Yanle Zhi[1,2*], Shengchao Wang[1,2], Haibo Zhang[2,3], Guimin Xue[2,3], Zhiqiang Zhang[1,2*]

1. Academy of Chinese Medical Sciences, Henan University of Chinese Medicine, Zhengzhou, 450045
2. Henan University of Chinese Medicine, Collaborative Innovation Center of Research and Development on the Whole Industry Chain of Yu-Yao, Henan, China
3. Engineering Technology Research Center of TCM Health Industry in Henan Province

Corresponding authors*

Yanle Zhi, Professor. Academy of Chinese Medical Sciences, Henan University of Chinese Medicine, Zhengzhou, 450045, Henan Province, China. Email: zhiy_l@hactcm.edu.cn

Zhiqiang Zhang, Professor. Academy of Chinese Medical Sciences, Henan University of Chinese Medicine, Zhengzhou, 450045, Henan Province, China. Email: zhiqiangzhang2022@hactcm.edu.cn

1.Materials and Methods

Gel retardation assays were performed to confirm the ability of P-CaO2@Au@OVA@Cu@DHPs to condense mRNA and provide protection from degradation. First, P-CaO2@Au@OVA@Cu@DHPs/mRNA complexes with 1 µg mRNA were prepared as described above at various N/P ratios from 4 to 32 in a final volume of 5 µL. Each of these complexes was mixed with 1 µL 6 × RNA loading buffer (Beyotime Biotechnology, Shanghai, China) and electrophoresed on a 1% (w/v) agarose gel for 45 min at 100 V. Then, mRNA retardation was visualized and photographed by ChemiDoc XRS imaging system (Tanon5200 Multi, Shanghai, China). In the second study, RNase A (Beyotime Biotechnology, Shanghai, China) was incubated with naked eGFP mRNA (1.0 µg) or the equivalent amount of mRNA complexed with P-CaO2@Au@OVA@Cu@DHPs at the N/P ratios of 16 and 32. After 10 min, the mixture was also analyzed by electrophoresis.

HEK-293T cells were transfected with P-CaO2@Au@OVA@Cu@DHPs/eGFP mRNA complexes. One day before transfection, cells were seeded in a 24-well plate at the density of 2 × 105 cells

per well. After 24 h, complete medium was replaced by serum-free medium. PVES/mRNA complexes at various N/P

ratios (1.5 μg mRNA/well) were added to cells. 4 h after transfection, the cell culture medium was replaced with fresh complete culture medium and the cells were incubated for another 20 h. Positive controls of transfection were performed with PEI 25 k/mRNA and Lipofectamine 3000/mRNA complexes according to the standard protocol. Negative control was performed with PEI 1.8 k/mRNA complexes at equivalent concentration.

1. Supplement Figures

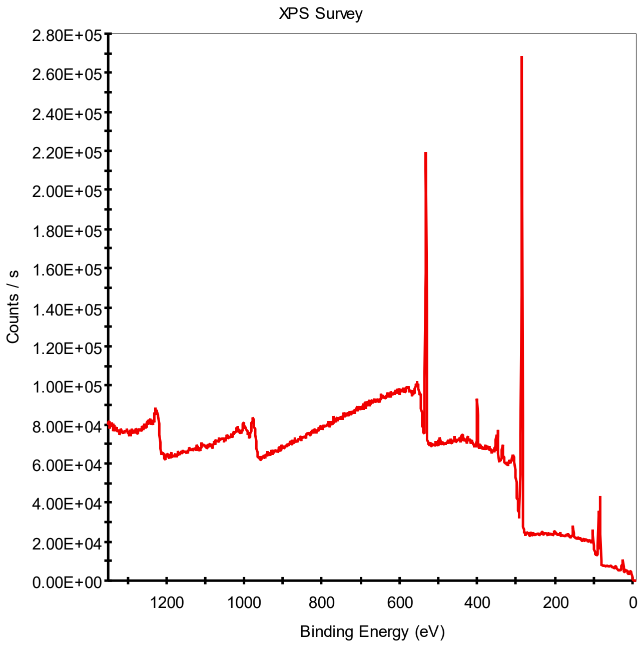

Figure S1 XRS of P-CaO_2-Au@OVA@Cu@DHPs

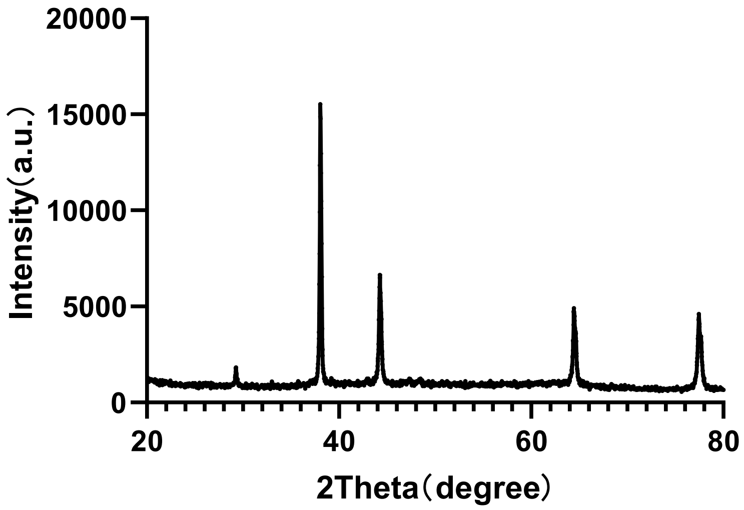

Figure S2 XRD of P-CaO_2-Au@OVA@Cu@DHPs

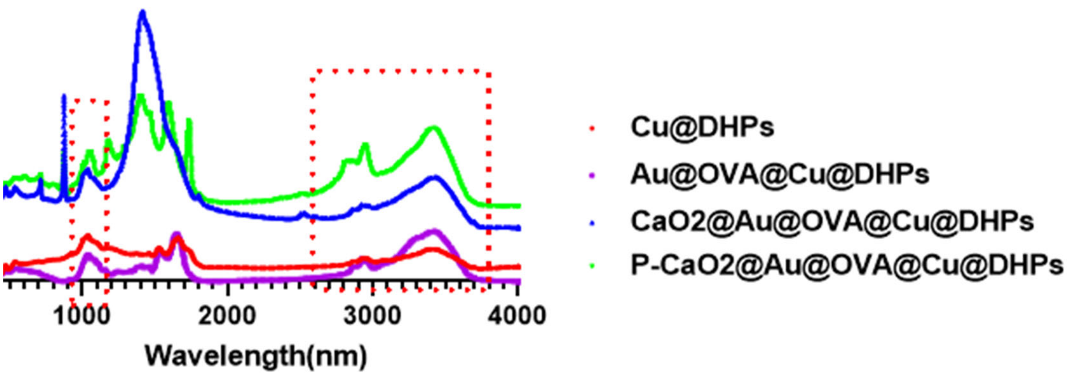

Figure S3. FTIR of Cu@DHPs,Au@OVA@ Cu@DHPs,

CaO_2-Au@OVA@Cu@DHPs and P-CaO_2-Au@OVA@Cu@DHPs

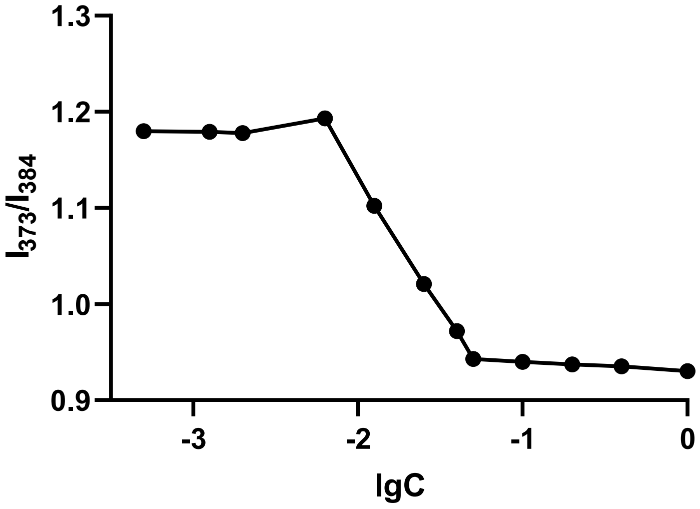

Figure S4.CMC of P-CaO_2-Au@OVA@Cu@DHPs

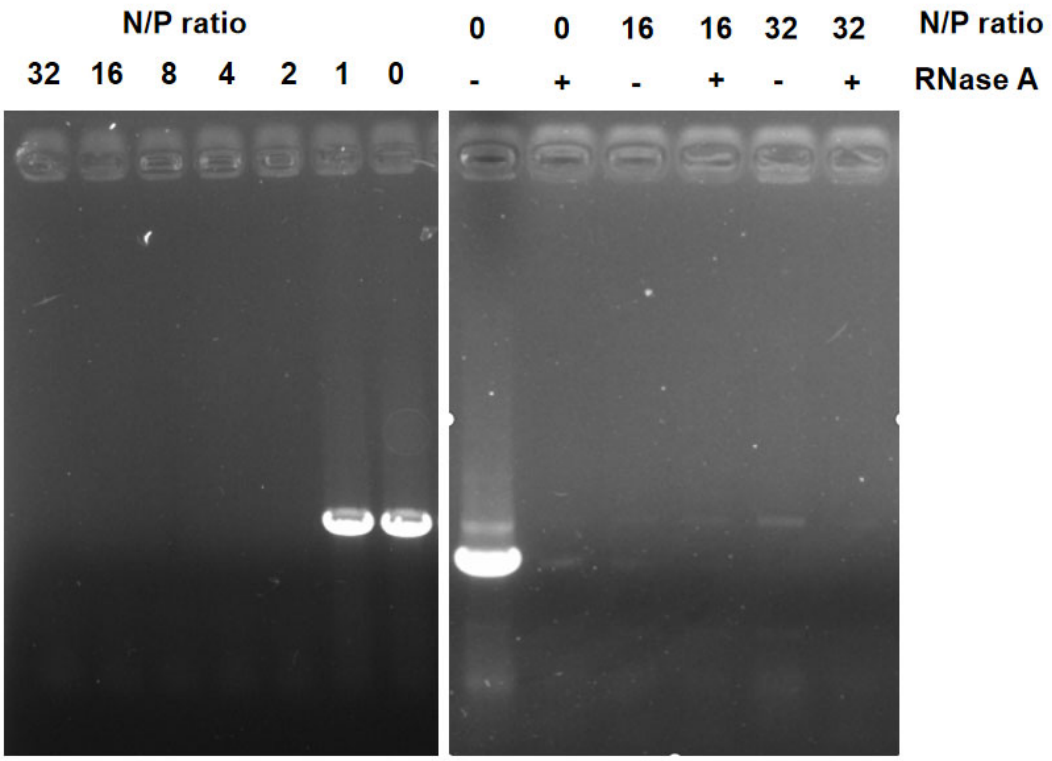

Figure S5.Gel retardation assays to detect condensation of mRNA into P-CaO_2-Au@OVA@Cu@DHPs at different N/P ratios (left) and protection efficiency from RNase A degradation at N/P ratios of 16 and 32 (right).

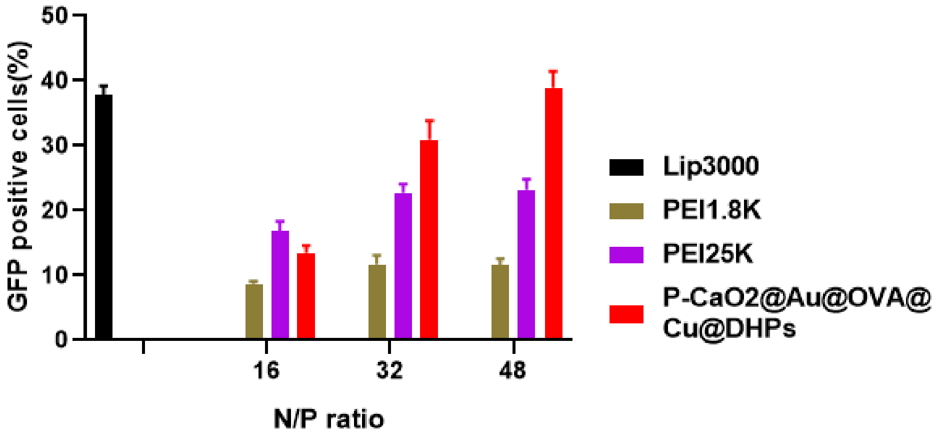

Figure.S6 In vitro P-CaO_2-Au@OVA@Cu@DHPs/mRNA complexes transfection and expression in HEK-293T cells. After 24 h transfection, expression of eGFP mRNA were analyzed by flow cytometry. eGFP expression efficiency was evaluated by the number of GFP positive cells

measured by flow cytometry. Lipofectamine 3000, PEI 25 k and PEI 1.8 k were used as controls. Data were shown as mean ± SEM.
